# Discovered Key CpG Sites by Analyzing DNA Methylation and Gene Expression in Breast Cancer Samples

**DOI:** 10.3389/fcell.2022.815843

**Published:** 2022-02-01

**Authors:** Yan-Ni Cao, Qian-Zhong Li, Yu-Xian Liu

**Affiliations:** ^1^ Laboratory of Theoretical Biophysics, School of Physical Science and Technology, Inner Mongolia University, Hohhot, China; ^2^ The State Key Laboratory of Reproductive Regulation and Breeding of Grassland Livestock, Inner Mongolia University, Hohhot, China

**Keywords:** gene expression, DNA methylation, correlation, breast cancer, molecular targets

## Abstract

Breast cancer is the most common cancer in the world, and DNA methylation plays a key role in the occurrence and development of breast cancer. However, the effect of DNA methylation in different gene functional regions on gene expression and the effect of gene expression on breast cancer is not completely clear. In our study, we computed and analyzed DNA methylation, gene expression, and clinical data in the TCGA database. Firstly, we calculated the distribution of abnormal DNA methylated probes in 12 regions, found the abnormal DNA methylated probes in down-regulated genes were highly enriched, and the number of hypermethylated probes in the promoter region was 6.5 times than that of hypomethylated probes. Secondly, the correlation coefficients between abnormal DNA methylated values in each functional region of differentially expressed genes and gene expression values were calculated. Then, co-expression analysis of differentially expressed genes was performed, 34 hub genes in cancer-related pathways were obtained, of which 11 genes were regulated by abnormal DNA methylation. Finally, a multivariate Cox regression analysis was performed on 27 probes of 11 genes. Three DNA methylation probes (cg13569051 and cg14399183 of *GSN*, and cg25274503 of *CAV2*) related to survival were used to construct a prognostic model, which has a good prognostic ability. Furthermore, we found that the cg25274503 hypermethylation in the promoter region inhibited the expression of the *CAV2*, and the hypermethylation of cg13569051 and cg14399183 in the 5′UTR region inhibited the expression of *GSN*. These results may provide possible molecular targets for breast cancer.

## Introduction

Breast cancer is the most common malignant tumor in women and the main cause of cancer deaths in women worldwide. There were more than 2 million new breast cancer patients and more than 620,000 patients who died of breast cancer in 2018 (Bray et al., 2018). Since 2004, the incidence of breast cancer has increased slightly at a rate of about 0.3% per year (Siegel et al., 2020). At present, despite the use of many advanced treatment technologies to improve survival, but the quality of life for patients is poor, and for most patients, the finding of disease is in the late stage, or metastasis occurs at the late stage of diagnosis (Punglia et al., 2007). Therefore, it is particularly important to study breast cancer.

DNA methylation is a heritable and reversible epigenetic modification that can regulate gene expression without changing the DNA sequence. It mainly occurs at CpG sites and is considered to be a goalkeeper for long-term stable regulation of gene expression (Cedar and Bergman, 2009). DNA methylation of different regions has both positive and negative correlations with gene expression in breast cancer (Győrffy et al., 2016). Many studies have reported that DNA methylation in the promoter region is negatively correlated with gene expression (Li et al., 2016; Janostiak et al., 2018). Studies have also found that DNA methylation is positively correlated with gene expression levels in the gene body region (Yang et al., 2014). It has been reported that DNA methylation may play a key role in the process of carcinogenesis by down-regulating the expression of tumor suppressor genes (Jones and Baylin, 2007). The hypermethylation in the promoter region of tumor suppressor genes is related to gene inactivation and transcriptional inhibition, and the hypermethylation of CpG islands (CGI) in the promoter region is considered to be one of the earliest and most frequent changes in cancer (Baylin, 2005; Wittenberger et al., 2014). There are some studies have shown that the hypomethylation of the enhancer region is closely related to up-regulation for gene expression in breast cancer (Jin et al., 2019). The hypomethylation in the promoter region is related to the activation of oncogenes and metastasis-promoting genes and has been verified to play an important role in the occurrence, development, and metastasis of cancer (Stefanska et al., 2011; Jones, 2012; Nilsson et al., 2014). Thus, it is necessary to study the effect of DNA methylation in different regions on gene expression.

Studies have shown that abnormal DNA methylation is considered to be a key factor leading to the carcinogenesis of various tumors, including breast cancer (Kulis and Esteller, 2010; Karsli-Ceppioglu et al., 2014). For example, *PSAT1* methylation is associated with HR-positive, lymph node-positive breast cancer, and invasive lobular cancer. *GNE* methylation is associated with HR-negative breast cancer, while *CXCL14* methylation is associated with HER2-positive breast cancer (Bu et al., 2013). Studies have also found that *DACT2* promoter methylation is related to advanced tumor staging (Borgonio-Cuadra et al., 2018). *CRY2* is an independent indicator that reduces the risk of metastasis and recurrence in ER+ breast cancer patients (Liu et al., 2017). *DFNA5* methylation shows strong potential as a biomarker for breast cancer detection and prognosis (Croes et al., 2018). Therefore, it is very meaningful to find key genes regulated by DNA methylation in breast cancer.

Although there have been substantial advances in breast cancer treatment, the treatment of breast cancer is still limited due to the lack of precise breast cancer molecular targets (Tang et al., 2018). In this study, to find molecular targets of DNA methylation that affect breast cancer prognosis. Firstly, the correlations between gene expression values and abnormal DNA methylation in different functional regions of four types of genes were computed and analyzed. Secondly, breast cancer may not be caused by the regulation of a single gene but by the joint regulation of multiple genes. To find candidate molecular targets and describe the correlation patterns between genes, we used weighted gene co-expression network analysis (WGCNA) to construct a co-expressed gene network. Based on the analyses of the correlation between the module gene and the clinical characteristics of the samples, two modules that were strongly related to cancer were obtained, and the hub genes were selected by analyzing the importance of the gene in the module. We analyzed the pathways of these hub genes and selected genes enriched in key pathways as the key genes for our research. Then by analyzing the genes whose absolute value of the correlation between expression value of key genes and DNA methylation of different sites was greater than or equal to 0.6, we found that our results verify that promoter methylation was negatively correlated with gene expression, and gene body region methylation was positively correlated with gene expression. Finally, a multivariate Cox regression analysis was performed on the 27 probes, and a regression model was constructed using three probes. Survival analysis shows that the prognostic performance of the model is good. Consequently, the three probes may be molecular targets related to methylation in breast cancer.

## Materials and Methods

### Data Sources

We downloaded the gene expression data [fragments per kilobase of exon model per million mapped fragments (FPKM) and COUNTS], DNA methylation data (HM450K), and clinical data (Supplementary Table S1) (hg38) of breast cancer and paracancerous tissues from the TCGA (The Cancer Genome Atlas) (https://tcga-data.nci.nih.gov/tcga/) database (Supplementary Table S2). We downloaded the human reference genome annotation file RefSeq gene (hg38) and the location file of CGI from UCSC (http://genome.ucsc.edu/). The position file of the enhancer was obtained from the FANTOM5 (Function Annotation of The Mammalian Genome) (https://fantom.gsc.riken.jp/5/) database.

### Data Preprocessing and Division of Different Regions

For gene annotation file, we retained 57,392 transcripts starting with the NM (the mature messenger RNA). We randomly reserve one of the transcripts with the same transcription start site (TSS), leaving 19,495 genes. Finally, the genes on chromosomes 1-22, X, and Y were retained, and a total of 19,484 genes encoding proteins were obtained for this study. We divided the genome into six regions {promoter (1,500 bp upstream and downstream of TSS), 5′UTR, exon, intron, 3′UTR, and intergenic region [from the transcription termination site (TTS) of one gene to the TSS of the next gene]}. In addition, we also selected the enhancer region and used the position of the enhancer to find the gene closest to it, and defined this gene as the target gene of the enhancer. After processing the location file of CGI, we obtained five regions [N_Shelf (2–4 kb upstream of CGI), N_Shore (0–2 kb upstream of CGI), CGI, S_Shore (0–2 kb downstream of CGI), and S_Shelf (2–4 kb downstream of CGI)]. Then we divided the promoter region into 30 windows in 100 bp, and each of the other 11 regions was divided into ten windows on average.

### Calculation of Differentially Methylated CpG Sites and Average DNA Methylation Level

First, we integrated the downloaded DNA methylation data of 789 breast cancer and 96 paracancerous samples into a matrix. Then we used the Limma package for differential analysis (Ritchie et al., 2015). Finally, we selected the probe of |Δβ|≥0.2,p<0.05 , adjusted *p*-value< 0.01, 14,855 hypermethylated probes and 11,056 hypomethylated probes were obtained (Supplementary Table S3). We collectively refer to hypermethylated probes and hypomethylated probes as abnormal DNA methylated probes (ADMPs).

$$\Delta \beta =\beta_{\omega ,c}-\beta_{\omega ,n}$$ (1)

here $\beta_{\omega ,c}$ denotes the methylation level of the ω -th CpG site (probe) in the cancer sample, the $\beta_{\omega ,n}$ denotes the methylation level of ω -th CpG site in the paracancerous sample.

We matched ADMPs to 12 different regions of the gene and calculated the DNA methylation level of each region for the gene. To better understand the abnormal DNA methylation characteristics of each region, we calculated the average DNA methylation level of each region according to the following formula:

$$\beta_{b}=\sum_{i=1}^{k}\beta_{b,i}/k$$

$$\beta_{b,i}=\sum_{\omega =1}^{m}\beta_{\omega }/m$$ (2)

here $\beta_{b}$ denotes the average DNA methylation level of the *b*th bin, $\beta_{b,i}$ denotes the methylation level of the *b*th bin in the *i*th gene, and *k* represents the number of genes whose DNA methylation level is not 0. $\beta_{\omega }$ denotes the DNA methylation level of the ω -th CpG site. *m* represents the number of probes falling into the *b*th bin of the *i*th gene.

### Analysis of Differentially Expressed Genes

We used the DESeq2 package to process gene expression data (Love et al., 2014). First, the expression data were integrated and normalized into a matrix, and log_2_FC (foldchange = cancer/normal) >1, *p* < 0.05, adjusted *p*-value< 0.05, was used as the threshold. Finally, 5,063 differentially expressed genes (DEGs) were obtained, of which 3,030 genes were up-regulated, and 2,033 genes were down-regulated (Supplementary Table S4).

### Selection of Co-Expressed Genes and Hub Genes

We used the WGCNA package to calculate the Pearson correlation coefficient between DEGs, and construct a similarity matrix. To better satisfy the structure of the scale-free network, the similarity matrix was transformed into a connectivity matrix through suitable soft thresholding. The topological overlap matrix (TOM) was calculated through the connectivity matrix, and finally, the degree of dissimilarity matrix was obtained through 1-TOM. Through the dissimilarity matrix, genes could be easily clustered to obtain different gene modules. When selecting the hub genes, first, we calculated the logarithm of the *p*-value after linear regression between the gene expression value and the clinical characteristics. It represents the relationship between gene expression and clinical characteristics in the module, that is, the Gene Significance (GS). If the absolute value of GS for a gene is greater, the biological significance of the gene is greater. Second, the Pearson correlation coefficient between the gene expression value and the characteristic gene of a given module was calculated, that is, the Module Membership (MM). The larger the MM value of a gene, the more important the gene is in the module (Langfelder and Horvath, 2008).

### Correlation Analysis

We used 75 breast cancer samples with DNA methylation and gene expression in both cancerous and para-cancerous tissues. Due to the characteristics of DNA methylation and gene expression data, we used Spearman correlation to calculate the correlation between DNA methylation and gene expression, as shown in Eq. 3.

$$r_{\omega }=1-\frac{6{\sum_{j=1}^{n}\left(rg_{\beta_{\omega ,j}}-rg_{e_{i,j,\omega }}\right)}^{2}}{n\left(n^{2}-1\right)}$$ (3)

here $rg_{\beta_{\omega ,j}}$ denotes the order of the DNA methylation value of ω -th probe in the *j*th sample, $rg_{e_{i,j,\omega }}$ denotes the order of the gene expression value of the *i*th gene where the ω -th probe is located for the *j*th sample, $r_{\omega }$ denotes the Spearman correlation coefficient between the DNA methylation value of the ω -th probe and the expression value of its gene.

### Prognostic Model Construction

We selected 11 genes because they are strongly correlated with tumor status, they are all enriched in pathways related to cancer, and their gene expression values are strongly correlated with DNA methylation. Then we used the methylation level of 27 CpG sites on the 11 genes and survival information to establish a multivariate Cox proportional hazard regression model, in which significant CpG sites were regarded as typical CpG sites. Finally, three methylation sites (cg25274503, cg13569051, and cg14399183) in the risk ratio model were determined. A risk score was established, the coefficient was weighted by the Cox model, and the risk score was calculated according to the following formula:

Risk Score=∑Coef×β (4)

where Coef represents the regression coefficient of the CpG site on the prognostic risk score, and β is the methylation level of CpG site.

## Results

### Genome-Wide DNA Methylation Analysis

**FIGURE 1:**
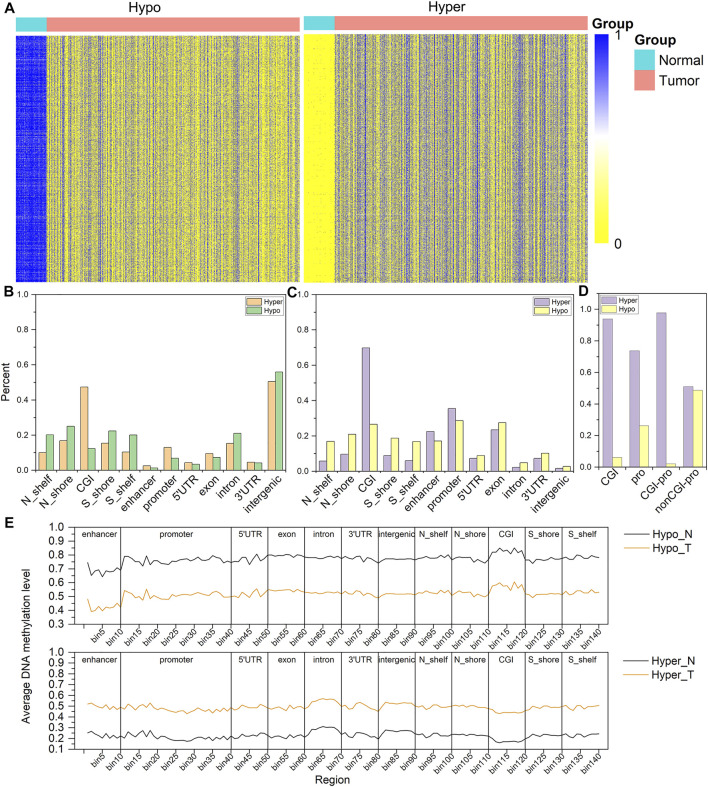
Distribution map of DNA methylation in the whole genome. **(A)** is the methylation value of hypomethylation (Hypo) and hypermethylation (Hyper) probes in different samples, the rows are the methylation values of each probe, and the columns are the different samples. **(B)** is the density distribution of DNA methylation probes in each functional region of the genome. **(C)** is the density distribution of DNA methylation probes in each functional region of the genome normalized by length. **(D)** is the distribution ratio diagram of the hyper and hypo methylation probes of the CGI and the promoter region, **(E)** is the distribution of average DNA methylation levels in 12 regions of ADMPs in paracancerous tissues. (N) and breast cancer tissues (T). The abscissa bin1-bin140 is divided into 12 regions by 11 vertical lines, and the upper coordinate is the name of each region.

To understand the distribution of ADMPs in the breast cancer genome, we calculated the difference in DNA methylation. The 14,855 hypermethylated probes and 11,056 hypomethylated probes were obtained (Figure 1A). The ADMPs were matched to 12 regions of the genome (Figures 1B,C,E). In both sides of CGI, intergenic regions, and intron regions, the degree of enrichment for hypomethylated probes is higher than that for hypermethylated probes (Figure 1B). The hypermethylated probes are highly enriched in CGI and promoter regions. Then we normalized the number of probes distributed in each region according to the length of each region, and the distribution was shown in Figure 1C. The enrichment degree of hypermethylated probes is higher than that of the hypomethylated probes in CGI, enhancer regions, and promoter regions. In other regions, the enrichment degree of hypomethylated probes is higher than that of hypermethylated probes. By comparing Figures 1B,C, we found that hypermethylated probes are more significantly enriched in CGI and promoter regions after normalization by length. We found that the hypermethylated probes in CGI and promoter regions are highly enriched both before and after normalization. So, we took the intersection between the hypermethylated probes in the CGI and the hypermethylated probes in the promoter region (Figure 1D) and found that most of the hypermethylated probes in the promoter region are located on the CGI. And we found that the number of hypermethylated probes is almost the same as the number of hypomethylated probes in the promoter region without CGI. In the promoter region with CGI, hypermethylated probes is as high as 97.87%. It can be speculated that the enrichment of hypermethylated probes in the promoter region is caused by the enrichment of CGI. Our results also confirmed that hypermethylated probes are mainly concentrated in CGI of the promoter regions.

We further explored the distribution of DNA methylation levels in the 12 regions (Figure 1E). In the paracancerous tissues, the DNA hypomethylated values are mainly concentrated in 0.65–0.85, the DNA hypermethylated values in each region are mainly concentrated in 0.15–0.3. In cancer tissues, the DNA hypomethylated values in each region are mainly concentrated in 0.4–0.55, the DNA hypermethylated values are mainly concentrated in 0.45–0.55. Regardless of whether the DNA methylation level of the cancer genome is a hypermethylated probe or a hypomethylated probe, the methylation level of each region of the gene is about 0.5.

### Analysis of the Correlation Between DNA Methylation and Gene Expression in Different Functional Regions of DEGs

**FIGURE 2:**
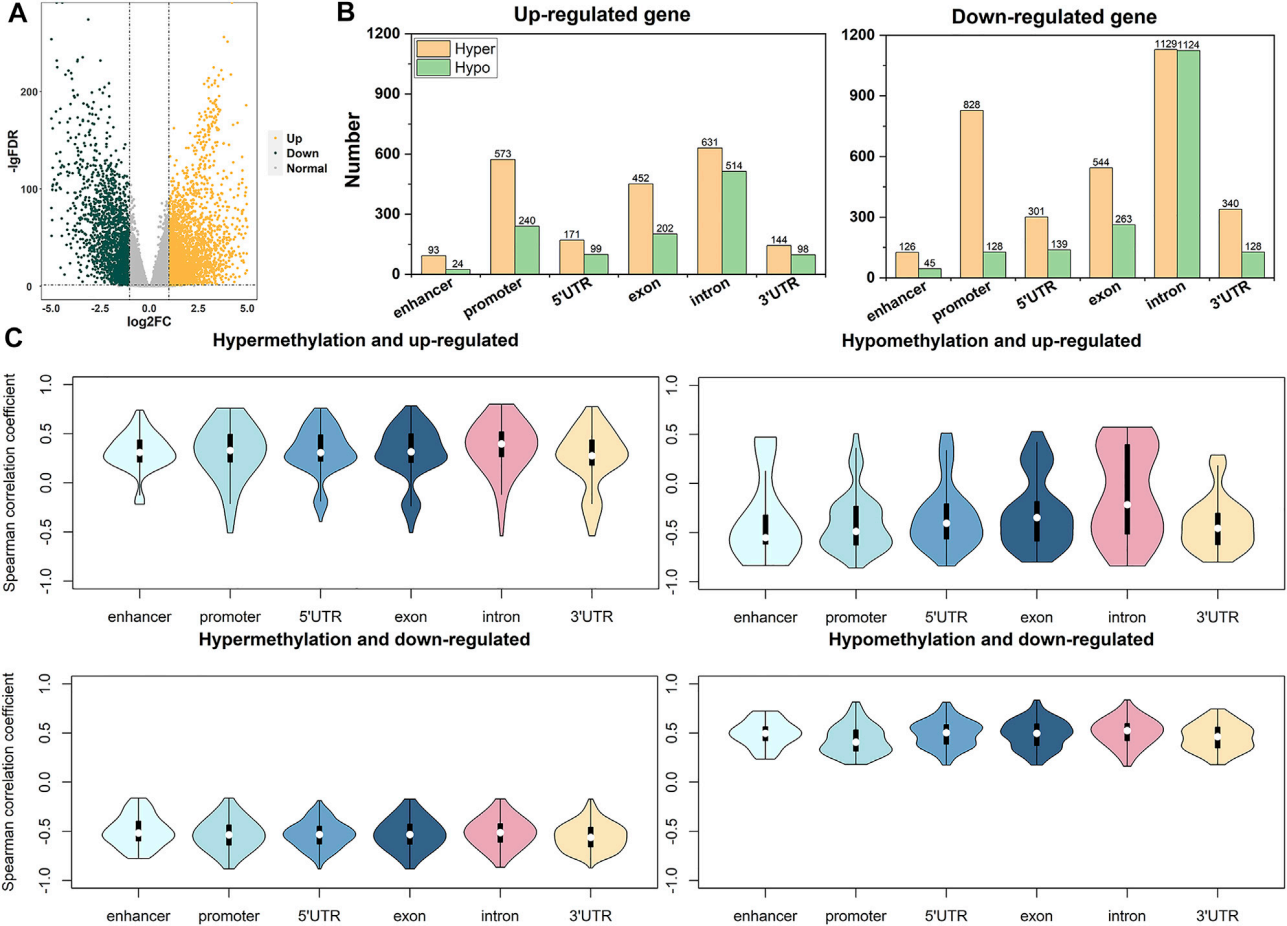
The distribution of correlation coefficients between gene expression and DNA methylation. **(A)** is a volcano map for screening DEGs. **(B)** is the enrichment number of DNA methylation probes in each region of up-regulated and down-regulated genes. **(C)** is the correlation between DNA methylation and gene expression in each region of the four types of genes.

To understand the regulatory effect of DNA methylation on gene expression, we calculated the DEGs between breast cancer tissues and paracancerous tissues, of which 3,030 genes are up-regulated, and 2,033 genes are down-regulated (Figure 2A). We computed the ADMPs in various functional regions for up-regulated genes and down-regulated genes, as shown in Figure 2B. The total number of ADMPs in up-regulated genes is less than that in down-regulated genes. The number of up-regulated genes is about 1.5 times that of down-regulated genes, which further shows that ADMPs like to be enriched in down-regulated genes. The number of probes enrichment in the 5′UTR and 3′UTR of the up-regulated genes is almost the same, and the same is true in the down-regulated genes. The number of hypermethylated probes in the promoter and exon regions of up-regulated genes is about twice the number of hypomethylated probes, and the same pattern is observed in the exons of down-regulated genes. However, the number of hypermethylated probes in the promoter region of down-regulated genes is 6.5 times that of hypomethylated probes, which is quite different from up-regulated genes. From the up- and down-regulation of gene expression and the hyper- and hypo-methylation of probes, it can be divided into four categories (hypermethylated up-regulated genes, hypomethylated up-regulated genes, hypermethylated down-regulated genes, and hypomethylated down-regulated genes). We calculated the Spearman correlation coefficients between the gene expression value and each CpG probe falling into the functional region of the differentially expressed gene in these four types of genes. The correlation coefficients with *p* < 0.05 were selected for display, as shown in Figure 2C. We can see that the expression of hypomethylated up-regulated genes and hypermethylated down-regulated genes are negatively correlated with the DNA methylation level of the probes. The expression of hypermethylated up-regulated genes and hypomethylated down-regulated genes are positively correlated with the DNA methylation level of the probes. In summary, the abnormal DNA methylation of down-regulated genes is highly enriched. The most obvious manifestations are the promoter region. Among them, the number of hypermethylated probes in the promoter region of down-regulated genes is much higher than that of hypomethylated probes.

### Discovery of Modular Genes Related to Breast Cancer and Screening of Hub Genes

**FIGURE 3:**
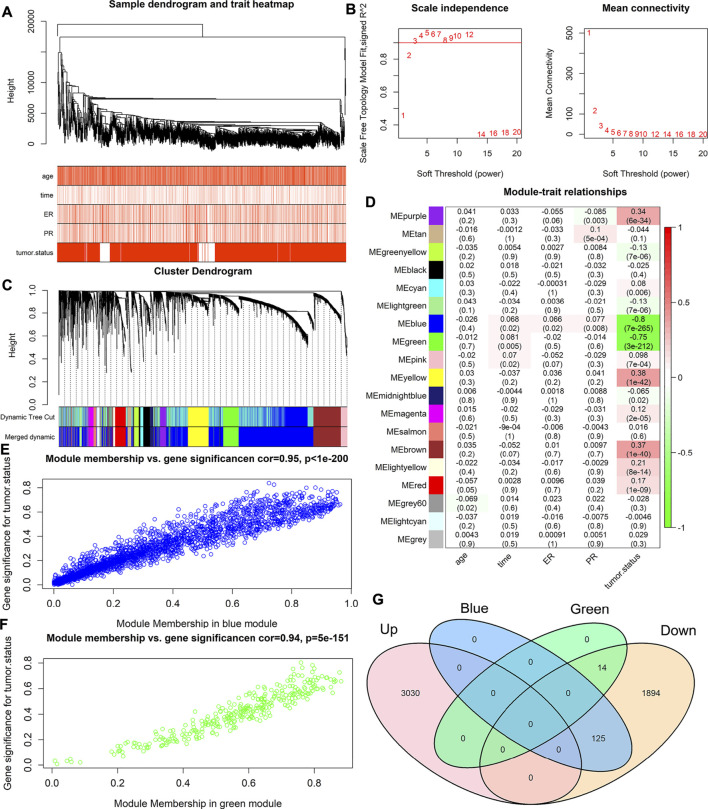
WGCNA analysis of DEGs. **(A)** is a sample clustering tree diagram. **(B)** is the scale-free index of various soft threshold power, and the average connection degree of various soft threshold power. **(C)** is the module gene color block of cluster analysis. The first line is the module generated by the first clustering, and the second line is the merged module (when the similarity between different modules reaches 0.8, they are merged into one module). **(D)** is the heat map of the correlation coefficient between module genes and clinical features. **(E)** is the scatter plot of the distribution of the Module Membership (MM) and the Gene Significance (GS) in the blue module. **(F)** is the scatter plot of the distribution of MM and GS in the green module. **(G)** is the Venn diagram of the hub gene and the expression up-regulated gene and the expression down-regulated gene.

The genes that affect breast cancer are not single, so we need to look for co-expressed gene clusters. We used 5,063 DEGs to find gene clusters related to breast cancer. First, we performed hierarchical clustering on all samples of DEGs and removed 14 outlier samples (Figure 3A). Then we constructed a scale-free network for the gene expression values of the remaining samples and chose three as the best soft threshold (Figure 3B). We set the minimum number of genes in each gene module to 30 and obtained 19 gene modules by clustering and merging similar modules (Figure 3C). At the same time, it can be seen that the gene expression is relatively independent between the modules (Supplementary Figure S1). Finally, the Pearson correlation analysis was carried out between 19 gene modules and clinical traits. Figure 3D shows that some module genes are strongly correlated with tumor status. Among them, MEblue and MEgreen show a strong negative correlation with tumor status, and the correlation coefficients are −0.8 (*p* = 7e-265) and −0.75 (*p* = 3e-212), respectively.

Because of the strong negative correlation between the above two gene modules and the tumor status, we calculated the GS and MM of each gene in blue and green modules. The results show that the MM and GS of genes in the blue module and the green module are highly linearly correlated (Figures 3E,F). Then we selected genes with MM value greater than or equal to 0.8 and GS value greater than or equal to 0.6 as hub genes that were highly correlated with clinical features (Supplementary Table S5). 14 hub genes were obtained in the green module, and 125 hub genes were obtained in the blue module, and these hub genes were all down-regulated (Figure 3G). In summary, it is indicated that the down-regulation of the 139 hub genes we obtained may be related to the occurrence of breast cancer.

### Pathway Analysis of Hub Gene

**FIGURE 4:**
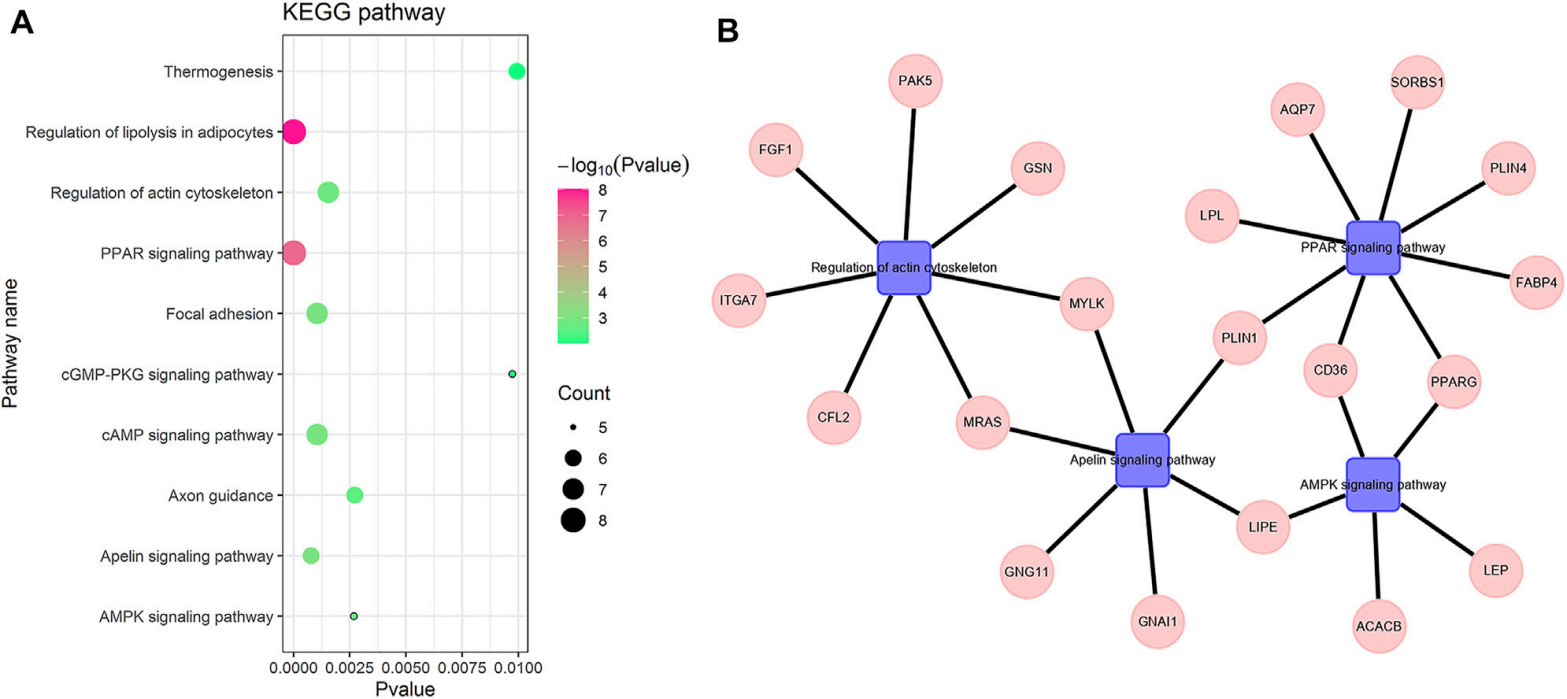
Hub gene-enriched KEGG pathway. **(A)** is the bubble chart of the hub gene-enriched pathway. **(B)** is the pathway-enriched gene, the square is the pathway name, and the circle is the gene name.

To understand the biological functions of the hub genes, we used GenCLiP3 (Wang et al., 2019) to analyze the pathways of these genes. We chose the KEGG pathway with *p*-value ≤ 0.01, Hit ≥ 5. It can be seen from Figure 4 that these genes are mainly enriched in the PPAR signaling pathway, which can regulate lipid metabolism, adipogenesis, maintain metabolic homeostasis and inflammatory gene expression, and have anti-cancer effects in a variety of tumors (Ahmadian et al., 2013). The hub genes are also enriched in the AMPK signaling pathway. Because of AMPK’s role in regulating energy homeostasis, AMPK is considered to be a potential target for developing new therapies for obesity, type 2 diabetes, metabolic syndrome, and cancer (Mihaylova and Shaw, 2011). The hub genes are also enriched in the Apelin signaling pathway, which is related to different key physiological processes, such as cell proliferation and energy metabolism regulation. On the other hand, it also involves a variety of pathologies, including diabetes, obesity, cardiovascular disease, and cancer (Antushevich and Wójcik, 2018). These genes are also enriched in the regulation of actin cytoskeleton pathway, which is mainly responsible for mediating various important cellular processes, including cell migration, proliferation, and survival (Saarikangas et al., 2010). These results indicate that hub genes are enriched in a variety of cancer-related pathways, further explaining the importance of these hub genes.

### Correlation Analysis Between Key Gene Expression and DNA Methylation

**FIGURE 5:**
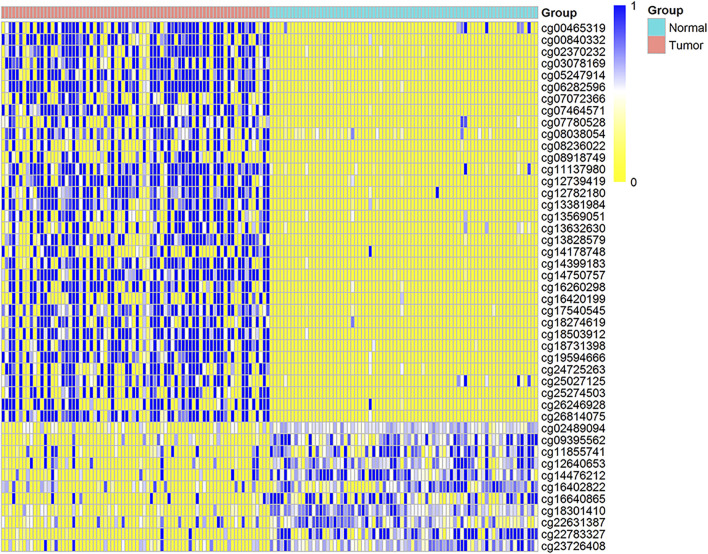
The DNA methylation distribution map of probes for 17 key gene, the distribution heat map of the DNA methylation value of 45 CpG sites in 150 matched cancers and paracancerous samples. The row represents the DNA methylation value of each CpG site, and the column represents each sample. Blue indicates the trend of DNA methylation value close to 1, and yellow indicates that the DNA methylation value is close to 0.

We have obtained 34 genes enriched in the key pathways and called them key genes. To understand the influence mechanism of abnormal DNA methylation on the key genes whose expression is down-regulated, we matched probes for abnormal DNA methylation corresponding to the 34 genes. In the end, only 17 key genes have ADMPs. Among them, 34 hypermethylated probes were matched on 14 genes, 11 hypomethylated probes were matched on six genes, and there were both hypermethylated probes and hypomethylated probes on three genes, *MGLL*, *FXYD1*, and *MYLK*. Figure 5 shows the DNA methylation value of the 45 probes on the cancer samples and matched paracancerous samples. We computed the Spearman correlation coefficients between the expression values of these 17 genes and the DNA methylation of 45 probes (Supplementary Table S6) and found that the hypomethylated probes for the key genes are mainly located in the intron and exon regions, and the DNA methylation of probes are positively correlated with gene expression. Then, we used 0.6 as the threshold, and the absolute value of the correlation coefficient greater than or equal to 0.6 was considered a significant correlation (*p* < 0.05). Finally, the DNA methylation values of the 27 probes are significantly correlated with the expression of 11 genes (Table 1). Among the 27 probes, the 25 probes are hypermethylated, and the expression of the 25 probes are significantly negatively correlated with their genes. Due to the overlap of gene annotation file regions, a probe may be in a different region. We see that most of these hypermethylated probes are located in the promoter, CGI, and intron, and the CGI are all contained in the promoter. Some hypermethylated probes are located in the 5′UTR region, the first exon, and enhancer regions. The cg16640865 and cg22783327 are hypomethylated, located in the 24th exon of *PDE2A* and the 5th intron of *FXYD1*, respectively. The results show that the CGI hypermethylation in the promoter for most key genes can inhibit gene expression. The hypomethylation of the 24th exon of *PDE2A* and the 5th intron of *FXYD1* also have an inhibiting effect on gene expression. This is consistent with the conclusion that the promoter methylation is negatively correlated with gene expression, and the methylation of the gene body region is positively correlated with gene expression.

**TABLE 1:** Table of ADMPs significantly correlated with gene expression.

Style	Gene	cg id	Chr	Position	Location	Spearman correlation coefficient	*p* value
hyper	*CAV2*	cg12739419	chr7	116500539	Promoter, 1st intron, S_shore	−0.7466	0
		cg16260298	chr7	116500288	Promoter, 1st intron, CGI	−0.6980	0
		cg25274503	chr7	116500074	Promoter, 1st intron, CGI	−0.6353	0
	*CFL2*	cg25027125	chr14	34713595	3rd intron, N_shore	−0.7061	0
	*FXYD1*	cg03078169	chr19	35138887	Promoter, N_shelf, N_shore	−0.6494	0
		cg05247914	chr19	35138797	Promoter, N_shelf	−0.7427	0
		cg07780528	chr19	35139430	Promoter, N_shelf	−0.6864	0
		cg17540545	chr19	35139451	Promoter, N_shelf, N_shore	−0.7986	0
		cg18503912	chr19	35139375	Promoter, N_shelf, N_shore	−0.8249	0
	*GNG11*	cg08038054	chr7	93921469	Promoter	−0.6857	0
	*GSN*	cg13569051	chr9	121289425	5′UTR, 10th intron	−0.6657	0
		cg13828579	chr9	121306136	12th intron	−0.6756	0
		cg14399183	chr9	121286030	5′UTR, 10th intron	−0.7008	0
	*LEP*	cg00840332	chr7	128241216	Promoter, CGI	−0.6269	9.38e-18
		cg07464571	chr7	128240948	Promoter, CGI	−0.6032	3.10e-16
		cg12782180	chr7	128240879	Promoter, CGI	−0.6268	9.46e-18
		cg13381984	chr7	128241291	Promoter, 5′UTR, 1st exon, CGI	−0.6302	5.57e-18
		cg19594666	chr7	128241227	Promoter, CGI	−0.6331	3.53e-18
		cg26814075	chr7	128241245	Promoter, CGI	−0.6136	6.97e-17
	*MGLL*	cg18274619	chr3	127776009	Enhancer, 4th intron	−0.7373	0
	*MYLK*	cg00465319	chr3	123620721	3rd intron	−0.6799	0
		cg18731398	chr3	123695886	16th intron	−0.7483	0
	*SEMA3G*	cg11137980	chr3	52435210	5′UTR, 1st exon	−0.6044	0
	*SORBS1*	cg02370232	chr10	95415608	18th intron	−0.6763	0
		cg06282596	chr10	95415722	18th intron	−0.6528	0
hypo	*FXYD1*	cg22783327	chr19	35142354	5th intron, N_shore	0.6777	0
	*PDE2A*	cg16640865	chr11	72590514	24th exon, CGI	0.6494	0

### The Construction and Evaluation of Prognostic Model

**FIGURE 6:**
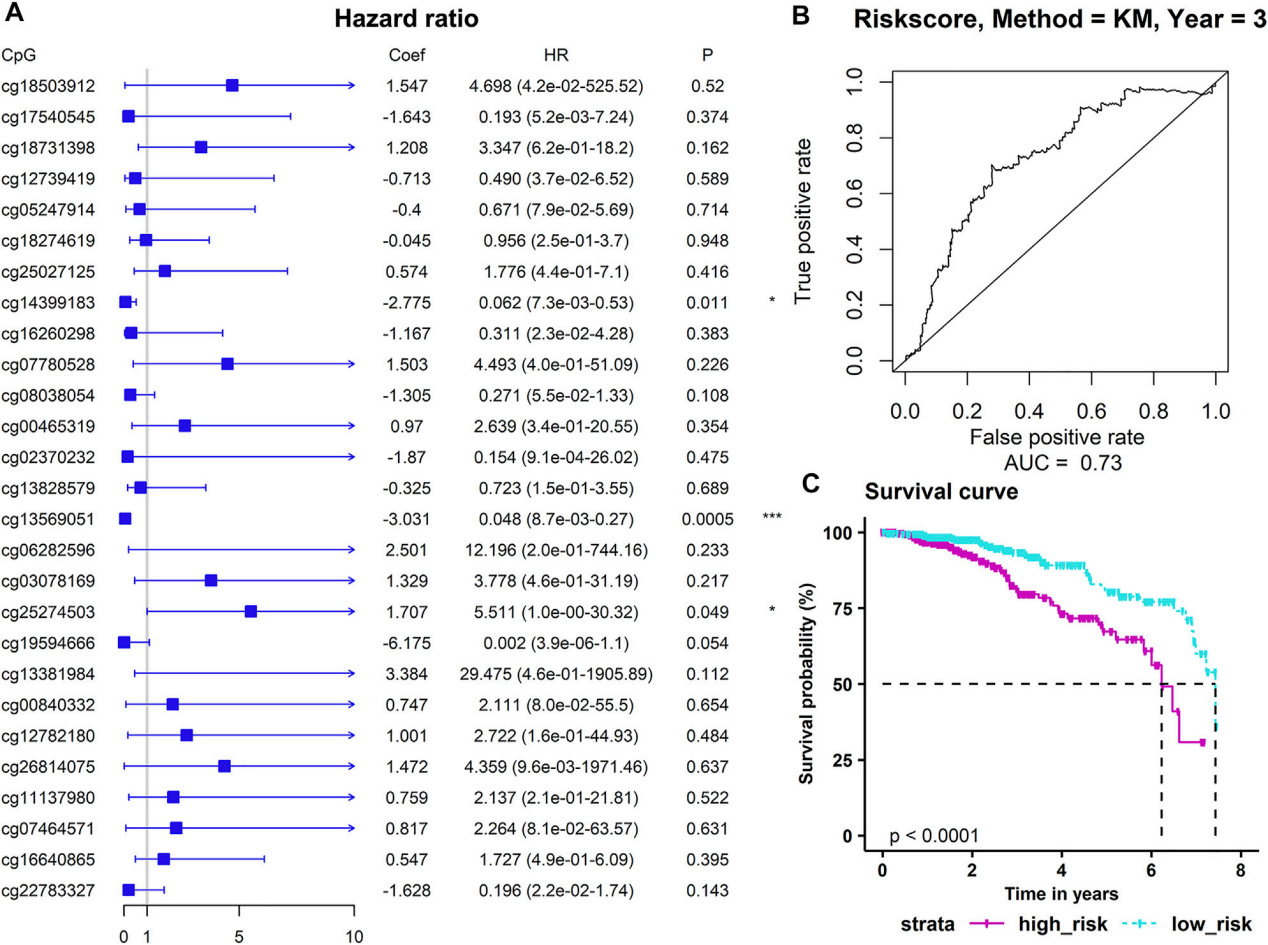
Construction of a prognostic model. **(A)** The results of multivariate Cox regression analysis. *p*-values were shown as: *p < 0.05; **p < 0.01; ***p < 0.001. **(B)** The sensitivity and specificity of the survival time of the sample. **(C)** The abscissa indicates the survival time (years), and the column indicates the survival rate.

We used the above 27 ADMPs that were significantly related to key hub gene expression for regression analysis (Figure 6A). Three ADMPs (cg13569051, cg14399183, and cg25274503) were obtained using multivariate Cox analysis, and a Cox proportional hazard model was constructed. The formula of our model is: Risk Score = −3.0311 × β value of cg13569051 + (−2.7747) × β value of cg14399183 + 1.7067 × β value of cg25274503. According to the risk scoring formula, we performed receiver operating characteristic (ROC) analysis on the risk score of each sample, and the area under the curve is AUC = 0.73 (Figure 6B), which shows that the model is good for the prognosis. Then, we divided the patients into a high-risk group and a low-risk group using the median of the risk score as the dividing line. Through Kaplan-Meier survival analysis, we found that the survival time of the high-risk group was significantly lower than that of the low-risk group (*p* < 0.0001) (Figure 6C).

**FIGURE 7:**
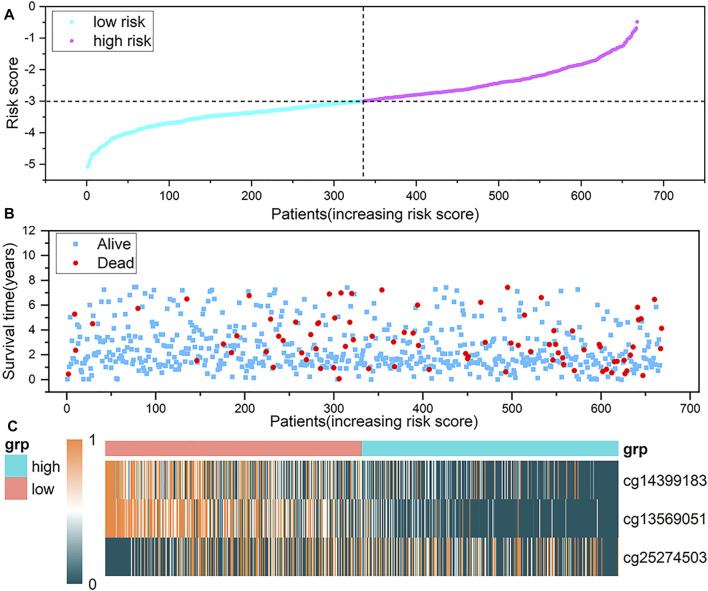
Analysis of risk scoring model. **(A)** represents the characteristic risk score distribution of the three methylation probes. **(B)** is a scatter plot of the patient’s survival time. **(C)** is the heat map of DNA methylation level, and a row represents a CpG probe, a column represents a patient. The three graphs have the same abscissa, and the abscissa is the patient in ascending order of risk score.

In addition, to observe whether the DNA methylation level of the probe in the model changes with the risk scoring model system, we sorted the samples according to the risk score. Figures 7A,B show a scatter plot of risk score distribution and patient status, where high risk is associated with more deaths. The heat map shows the methylation status of the three methylation probes between the high-risk group and the low-risk group (Figure 7C). The methylation levels of cg14399183 and cg13569051 decrease as the risk increases. The methylation level of cg25274503 increases with increasing risk. The cg14399183 and cg13569051 probes located in the 5′UTR of the *GSN* gene are protective factors for breast cancer, while the cg25274503 probe located in the promoter of the *CAV2* gene is a risk factor for breast cancer.

### DNA Methylation of *CAV2* and *GSN*

**FIGURE 8:**
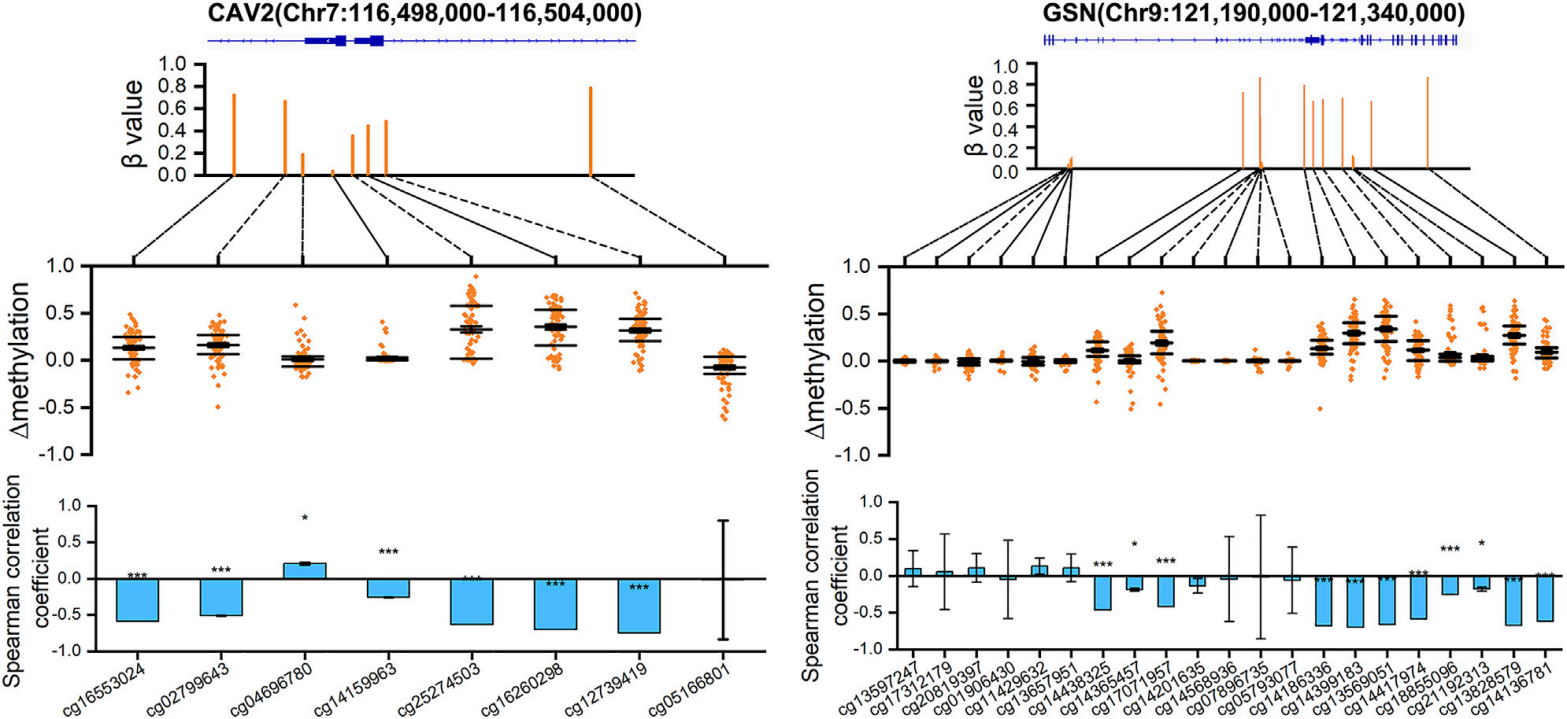
DNA methylation distribution map of *CAV2* and *GSN*. The scatter graph shows the Δβ level, the histogram shows the correlation coefficient between the DNA methylation of each probe and the gene expression value (*p*-values were shown as: *p < 0.05; **p < 0.01; ***p < 0.001).

According to the above analysis, we can see the importance of *CAV2* and *GSN* for breast cancer. Therefore, we visualized the DNA methylation values of all eight probes on *CAV2* and all 21 probes on *GSN* (*n* = 75) (Figure 8). We calculated the Δβ value of each probe in the 75 samples and determined that the probes on *CAV2* and *GSN* genes are hypermethylated probes except for the median of Δβ about 0. Then we calculated the correlation between the DNA methylation value of each probe and the gene expression level. We see that all hypermethylated probes are significantly negatively correlated with the down-regulated *CAV2* and *GSN* in breast cancer. These results indicate that the down-regulation of the two genes, *CAV2* and *GSN*, is caused by hypermethylation of important DNA methylation sites.

## Discussion

In this study, based on analysis of the correlation between abnormal DNA methylation in six different regions and gene expression of DEGs, co-expression analysis, and KEGG pathway analysis, 34 key hub genes co-expressed and strongly correlated with cancer status were obtained. The 27 ADMPs that were significantly related to gene expression were obtained. Based on three methylation probes (cg13569051, cg14399183, and cg25274503) in the *CAV2* and *GSN* genes, a risk scoring model with good prognostic performance was constructed. It is further confirmed that the three probes can be used as molecular targets for breast cancer.

Our results showed that in breast cancer samples, the hypermethylation of cg25274503 in the promoter was significantly negatively correlated with the down-regulation of *CAV2*. This rule is also reflected in other studies. For example, the hypermethylation of CGI silences the *CAV2* gene, which can be used as an obvious marker of breast cancer (Uehiro et al., 2016). At the same time, the hypermethylation of cg13569051 and cg14399183 in the 5′UTR was significantly negatively correlated with the down-regulation of *GSN*. Similar findings have been reported in previous studies. For example, *GSN* is down-regulated in gastric cancer cell lines, and promoter DNA methylation is involved in this process (Wang et al., 2017). In addition, the *CAV1* gene is highly methylated and lowly expressed (Li et al., 2016). In ER breast cancer patients, *FOXA1* hypermethylation is associated with the down-regulation of gene expression (Espinal et al., 2017). The silencing of gene expression by *PAQR3* promoter hypermethylation may play an important role in breast cancer (Nowak et al., 2017). These reflect the negative correlation between DNA methylation in the promoter region and gene expression. However, in previous studies on breast cancer, there was almost no discovery of the relationship between the DNA methylation of cg25274503 and *CAV2* expression and the DNA methylation of cg13569051 and cg14399183 and the *GSN* expression.

In fact, *CAV2* is a gene encoding caveolin 2, which is involved in basic cell functions, including signal transduction, lipid metabolism, control of cell growth, and apoptosis; it may have tumor suppressor effects (Fujimoto et al., 2001). In all types of lung cancer, *CAV2* is dysregulated at the RNA and protein levels (Wikman et al., 2004). Experiments have verified that the *CAV2* gene transcription is down-regulated in mice and humans with obstructive bladder disease (Thangavel et al., 2019). Studies have confirmed that compared with the corresponding normal tissues, the mRNA level of *CAV2* in human breast cancer tissues is significantly down-regulated (*p* < 0.001) (Sagara et al., 2004). *GSN* is an actin binding protein, a key regulator of actin filament assembly and disassembly, and is involved in cell movement, shape, and metabolism (Feldt et al., 2019). Studies have shown that *GSN* gene transcription is down-regulated in breast cancer of humans and some animals, and the activation of *GSN* may be a protective factor in the treatment of cancer cells against cancer (Mielnicki et al., 1999; Shahrokh et al., 2019). The secreted *GSN* inhibits the invasion and migration of colon cancer cells (Chen et al., 2019). The expression of *GSN* in bladder cancer is higher than that in normal tissues, and the prognosis of bladder cancer patients whose gene expression of *GSN* is up-regulated is worse (Yang et al., 2020). *GSN* is overexpressed in HCC tissues, and high *GSN* expression is significantly associated with advanced Edmondson grade, encapsulation, and multiple tumors (Zhang et al., 2020). These different studies show that the role of *GSN* in cancer depends on the type of cancer studied. It has been suggested that *GSN* has both the functions of tumor suppressor genes and oncogenes (Feldt et al., 2019). It can be seen from the above research that *CAV2* and *GSN* have important roles in a variety of cancers. Our study also found that *CAV2* and *GSN* were down-regulated in breast cancer, which is consistent with the results of previous studies.

In summary, our research has found two key genes (*CAV2* and *GSN*) related to breast cancer that may be regulated by DNA methylation and discovered three DNA methylation probes (cg13569051, cg14399183, and cg25274503). The risk scoring model was constructed by the three probes has a good prognostic ability. Therefore, these DNA methylation probes may be used as molecular targets for the prognosis of breast cancer.

## Data Availability

Publicly available datasets were analyzed in this study. This data can be found here: Gene expression data, DNA methylation data and clinical data from TCGA database (https://tcga-data.nci.nih.gov/tcga/); the genomic data from the UCSC database (http://genome.ucsc.edu/cgi-bin/hgTables); Ensembl (http://www.ensembl.org/Homo_sapiens/); the location file of CGI from UCSC (http://genome.ucsc.edu/); the location file of an enhancer from the FANTOM5 (Function Annotation of The Mammalian Genome) (https://fantom.gsc.riken.jp/5/).

## Abbreviations

ADMPs, abnormal DNA methylated probes; AUC, area under the receiver operating characteristic curve; CGI, CpG islands; DEGs, differentially expressed genes; FPKM, fragments per kilobase of exon model per million mapped fragments; GS, Gene Significance; MM, Module Membership; ROC, receiver operating characteristic; TSS, transcription start site; TTS, transcription termination site.
