# Supplementary material for: Discovered Key CpG Sites by Analyzing DNA Methylation and Gene Expression in Breast Cancer Samples
Source: Front Cell Dev Biol. 2022 Feb 1;10:815843. doi: 10.3389/fcell.2022.815843 (PMC8844453; doi:10.3389/fcell.2022.815843)
Supplement: Supplementary file 3 [file Table1.DOCX]

Supplementary Material

## Supplementary Figures


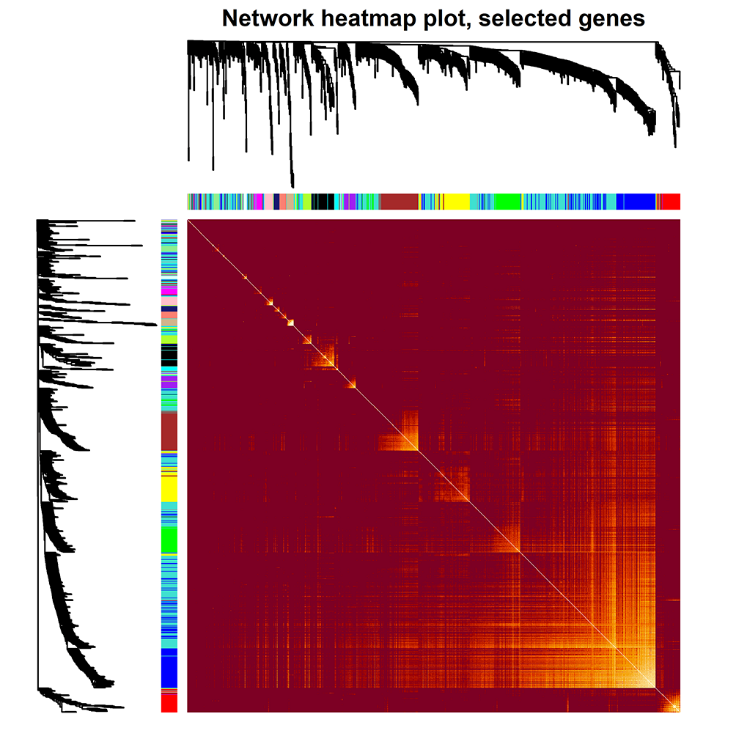


**Figure S1.** The heat map depicts the degree of dissimilarity matrix (1-TOM) between all modules included in the analysis. In the heat map, each row and each column correspond to a gene. Darker colors represent low overlap, and gradually lighter colors represent increased overlap. The light-colored squares along the diagonal correspond to the modules. The gene tree diagram and module allocation are shown on the left and top.

# Supplementary Tables

**Table S1.** Clinical information of breast cancer patients in this study.

| **Clinical character** | | **Number** | **Percent (%)** |
| --- | --- | --- | --- |
| Age | <=50 | 298 | 27.16 |
|  | >50 | 799 | 72.84 |
| Gender | Male | 12 | 1.09 |
|  | Female | 1085 | 98.51 |
| Pathologic M | M0 | 912 | 83.14 |
|  | M1 | 22 | 2.01 |
|  | Unknown | 163 | 14.86 |
| Pathologic N | N0 | 516 | 47.04 |
|  | N1 | 364 | 33.18 |
|  | N2 | 120 | 10.94 |
|  | N3 | 77 | 7.02 |
|  | Unknown | 20 | 1.82 |
| Pathologic T | T1 | 281 | 25.62 |
|  | T2 | 635 | 57.89 |
|  | T3 | 138 | 12.58 |
|  | T4 | 40 | 3.65 |
|  | Unknown | 3 | 0.27 |
| Pathologic stage | Ⅰ | 183 | 16.68 |
|  | Ⅱ | 621 | 56.61 |
|  | Ⅲ | 249 | 22.70 |
|  | Ⅳ | 220 | 20.05 |
|  | Unknown | 24 | 2.19 |
| Vital status | Dead | 152 | 13.86 |
|  | Alive | 945 | 86.14 |

**Table S5.** The Module Membership (MM) and Gene Significance (GS) values of 139 hub genes.

| **No.** | **Gene** | **Module** | **MM** | **GS** | **No.** | **Gene** | **Module** | **MM** | **GS** |
| --- | --- | --- | --- | --- | --- | --- | --- | --- | --- |
| 1 | SCN2B | green | 0.836 | 0.685 | 57 | MRAS | blue | 0.802 | 0.619 |
| 2 | TP63 | green | 0.851 | 0.612 | 58 | GPD1 | blue | 0.930 | 0.635 |
| 3 | CLDN19 | green | 0.865 | 0.661 | 59 | TUSC5 | blue | 0.935 | 0.656 |
| 4 | FGF1 | green | 0.824 | 0.697 | 60 | FP325317.1 | blue | 0.922 | 0.634 |
| 5 | NDRG2 | green | 0.802 | 0.625 | 61 | ABCA9 | blue | 0.834 | 0.735 |
| 6 | CLDN11 | green | 0.811 | 0.683 | 62 | ANKRD53 | blue | 0.851 | 0.648 |
| 7 | DST | green | 0.877 | 0.726 | 63 | PQLC2L | blue | 0.851 | 0.609 |
| 8 | MAML2 | green | 0.825 | 0.606 | 64 | ABCA8 | blue | 0.921 | 0.732 |
| 9 | KLHL29 | green | 0.839 | 0.687 | 65 | ROBO4 | blue | 0.808 | 0.668 |
| 10 | FAM126A | green | 0.860 | 0.671 | 66 | NPR1 | blue | 0.946 | 0.707 |
| 11 | MYLK | green | 0.850 | 0.672 | 67 | LEP | blue | 0.844 | 0.604 |
| 12 | PAK5 | green | 0.882 | 0.658 | 68 | PREX2 | blue | 0.835 | 0.620 |
| 13 | SAMD5 | green | 0.861 | 0.652 | 69 | AKAP12 | blue | 0.858 | 0.635 |
| 14 | KY | green | 0.807 | 0.666 | 70 | GSN | blue | 0.866 | 0.728 |
| 1 | PLIN4 | blue | 0.880 | 0.619 | 71 | EBF1 | blue | 0.949 | 0.707 |
| 2 | PLIN1 | blue | 0.957 | 0.656 | 72 | FHL1 | blue | 0.861 | 0.607 |
| 3 | ANTXR2 | blue | 0.878 | 0.637 | 73 | CHRDL1 | blue | 0.938 | 0.722 |
| 4 | CDO1 | blue | 0.913 | 0.661 | 74 | ECM2 | blue | 0.896 | 0.636 |
| 5 | CFL2 | blue | 0.833 | 0.717 | 75 | SPTBN1 | blue | 0.896 | 0.681 |
| 6 | CA4 | blue | 0.860 | 0.716 | 76 | HCAR2 | blue | 0.890 | 0.630 |
| 7 | DDR2 | blue | 0.908 | 0.644 | 77 | LDB2 | blue | 0.827 | 0.765 |
| 8 | CLMP | blue | 0.916 | 0.669 | 78 | DMGDH | blue | 0.912 | 0.632 |
| 9 | LIPE | blue | 0.932 | 0.631 | 79 | DTX1 | blue | 0.831 | 0.603 |
| 10 | GLYAT | blue | 0.915 | 0.649 | 80 | CCDC69 | blue | 0.920 | 0.651 |
| 11 | ITIH5 | blue | 0.910 | 0.700 | 81 | MCAM | blue | 0.892 | 0.632 |
| 12 | MYZAP | blue | 0.885 | 0.660 | 82 | BHMT2 | blue | 0.877 | 0.601 |
| 13 | GNAI1 | blue | 0.882 | 0.656 | 83 | GPR146 | blue | 0.902 | 0.688 |
| 14 | PLSCR4 | blue | 0.817 | 0.750 | 84 | RDH5 | blue | 0.942 | 0.653 |
| 15 | FABP4 | blue | 0.930 | 0.644 | 85 | AQP7 | blue | 0.924 | 0.629 |
| 16 | MTURN | blue | 0.891 | 0.663 | 86 | KIAA0408 | blue | 0.908 | 0.636 |
| 17 | KCNIP2 | blue | 0.931 | 0.627 | 87 | SVEP1 | blue | 0.869 | 0.727 |
| 18 | PLXNA4 | blue | 0.900 | 0.616 | 88 | PALMD | blue | 0.930 | 0.739 |
| 19 | SYNE3 | blue | 0.939 | 0.670 | 89 | CD300LG | blue | 0.937 | 0.771 |
| 20 | ACSM5 | blue | 0.907 | 0.613 | 90 | CAV2 | blue | 0.923 | 0.766 |
| 21 | BTNL9 | blue | 0.921 | 0.738 | 91 | ARHGAP20 | blue | 0.849 | 0.801 |
| 22 | HSPB7 | blue | 0.862 | 0.606 | 92 | GOLGA8M | blue | 0.815 | 0.620 |
| 23 | EBF3 | blue | 0.877 | 0.655 | 93 | CAV1 | blue | 0.951 | 0.763 |
| 24 | FOXN3 | blue | 0.846 | 0.648 | 94 | FZD4 | blue | 0.911 | 0.651 |
| 25 | GPAM | blue | 0.890 | 0.608 | 95 | LRRN4CL | blue | 0.841 | 0.755 |
| 26 | HEPACAM | blue | 0.938 | 0.643 | 96 | EHD2 | blue | 0.878 | 0.658 |
| 27 | SH3D19 | blue | 0.892 | 0.716 | 97 | PDE2A | blue | 0.899 | 0.785 |
| 28 | SLC35G2 | blue | 0.853 | 0.623 | 98 | MYOM1 | blue | 0.864 | 0.632 |
| 29 | ASPA | blue | 0.923 | 0.709 | 99 | FERMT2 | blue | 0.906 | 0.654 |
| 30 | ACACB | blue | 0.895 | 0.619 | 100 | EPAS1 | blue | 0.873 | 0.678 |
| 31 | LPL | blue | 0.918 | 0.629 | 101 | HSD17B13 | blue | 0.900 | 0.652 |
| 32 | RBP4 | blue | 0.902 | 0.624 | 102 | ACO1 | blue | 0.922 | 0.604 |
| 33 | PLPP3 | blue | 0.802 | 0.756 | 103 | ADGRF5 | blue | 0.865 | 0.666 |
| 34 | PDZD2 | blue | 0.870 | 0.626 | 104 | SIK2 | blue | 0.938 | 0.688 |
| 35 | ADRB2 | blue | 0.826 | 0.729 | 105 | FAM149A | blue | 0.830 | 0.654 |
| 36 | MGLL | blue | 0.827 | 0.601 | 106 | SEMA3G | blue | 0.844 | 0.663 |
| 37 | GYG2 | blue | 0.929 | 0.621 | 107 | TNS1 | blue | 0.948 | 0.696 |
| 38 | TMEM37 | blue | 0.891 | 0.623 | 108 | TMEM132C | blue | 0.930 | 0.663 |
| 39 | TIMP4 | blue | 0.876 | 0.600 | 109 | GHR | blue | 0.872 | 0.621 |
| 40 | KLB | blue | 0.898 | 0.612 | 110 | GNG11 | blue | 0.806 | 0.706 |
| 41 | CORO2B | blue | 0.835 | 0.732 | 111 | SORBS1 | blue | 0.936 | 0.662 |
| 42 | VWF | blue | 0.822 | 0.631 | 112 | FAM13A | blue | 0.836 | 0.718 |
| 43 | TLN2 | blue | 0.866 | 0.633 | 113 | CNRIP1 | blue | 0.838 | 0.614 |
| 44 | PPARG | blue | 0.924 | 0.654 | 114 | CIDEC | blue | 0.931 | 0.638 |
| 45 | HSPB6 | blue | 0.951 | 0.687 | 115 | GALNT15 | blue | 0.823 | 0.637 |
| 46 | NMT2 | blue | 0.811 | 0.627 | 116 | SLIT3 | blue | 0.810 | 0.727 |
| 47 | SDPR | blue | 0.879 | 0.802 | 117 | G0S2 | blue | 0.859 | 0.601 |
| 48 | FXYD1 | blue | 0.828 | 0.702 | 118 | LVRN | blue | 0.913 | 0.606 |
| 49 | ITGA7 | blue | 0.941 | 0.656 | 119 | CAT | blue | 0.890 | 0.655 |
| 50 | SGK2 | blue | 0.838 | 0.602 | 120 | GPX3 | blue | 0.842 | 0.614 |
| 51 | ANO6 | blue | 0.840 | 0.621 | 121 | MRAP | blue | 0.896 | 0.633 |
| 52 | CD34 | blue | 0.810 | 0.725 | 122 | GPBAR1 | blue | 0.830 | 0.612 |
| 53 | PTRF | blue | 0.800 | 0.684 | 123 | SLC19A3 | blue | 0.895 | 0.610 |
| 54 | NIPSNAP3B | blue | 0.820 | 0.617 | 124 | AOC3 | blue | 0.963 | 0.664 |
| 55 | PPP1R1A | blue | 0.875 | 0.604 | 125 | PCOLCE2 | blue | 0.902 | 0.635 |
| 56 | CD36 | blue | 0.935 | 0.668 |  |  |  |  |  |

**Table S6.** Table of 45 ADMPs associated with the key gene expression.

| Gene | cg | Chr. | Position | Location | r | p value | Style |
| --- | --- | --- | --- | --- | --- | --- | --- |
| CAV2 | cg12739419 | chr7 | 116500539 | Promoter, intron | -0.7466 | 0 | Hyper |
|  | cg16260298 | chr7 | 116500288 | Promoter, intron | -0.6980 | 0 | Hyper |
|  | cg25274503 | chr7 | 116500074 | Promoter, intron | -0.6353 | 0 | Hyper |
| CFL2 | cg25027125 | chr14 | 34713595 | intron | -0.7061 | 0 | Hyper |
| FXYD1 | cg03078169 | chr19 | 35138887 | promoter | -0.6494 | 0 | Hyper |
|  | cg05247914 | chr19 | 35138797 | promoter | -0.7427 | 0 | Hyper |
|  | cg07780528 | chr19 | 35139430 | promoter | -0.6864 | 0 | Hyper |
|  | cg17540545 | chr19 | 35139451 | promoter | -0.7986 | 0 | Hyper |
|  | cg18503912 | chr19 | 35139375 | promoter | -0.8249 | 0 | Hyper |
| GNG11 | cg08038054 | chr7 | 93921469 | promoter | -0.6857 | 0 | Hyper |
|  | cg08236022 | chr7 | 93921702 | promoter | -0.5621 | 0 | Hyper |
| GSN | cg13828579 | chr9 | 121306136 | intron | -0.6756 | 0 | Hyper |
|  | cg13569051 | chr9 | 121289425 | 5'UTR, intron | -0.6657 | 0 | Hyper |
|  | cg14399183 | chr9 | 121286030 | 5'UTR, intron | -0.7008 | 0 | Hyper |
| ITGA7 | cg24725263 | chr12 | 55707544 | intron | -0.4366 | 3.29e-08 | Hyper |
| LEP | cg13381984 | chr7 | 128241291 | 5'UTR, exon | -0.6302 | 5.57e-18 | Hyper |
|  | cg00840332 | chr7 | 128241216 | promoter | -0.6269 | 9.38e-18 | Hyper |
|  | cg07464571 | chr7 | 128240948 | promoter | -0.6032 | 3.10e-16 | Hyper |
|  | cg12782180 | chr7 | 128240879 | promoter | -0.6268 | 9.46e-18 | Hyper |
|  | cg13381984 | chr7 | 128241291 | promoter | -0.6302 | 5.57e-18 | Hyper |
|  | cg19594666 | chr7 | 128241227 | promoter | -0.6331 | 3.53e-18 | Hyper |
|  | cg26814075 | chr7 | 128241245 | promoter | -0.6136 | 6.97e-17 | Hyper |
| LPL | cg07072366 | chr8 | 19940494 | enhancer, Promoter, intron | -0.5526 | 0 | Hyper |
|  | cg16420199 | chr8 | 19940454 | enhancer | -0.4193 | 1.24e-07 | Hyper |
|  | cg08918749 | chr8 | 19939934 | Promoter, intron | -0.4175 | 1.41e-07 | Hyper |
|  | cg16420199 | chr8 | 19940454 | Promoter, intron | -0.4193 | 1.24e-07 | Hyper |
| MGLL | cg18274619 | chr3 | 127776009 | Enhancer, intron | -0.7373 | 0 | Hyper |
|  | cg14750757 | chr3 | 127787761 | intron | -0.5227 | 0 | Hyper |
| MYLK | cg18731398 | chr3 | 123695886 | intron | -0.7483 | 0 | Hyper |
|  | cg00465319 | chr3 | 123620721 | intron | -0.6799 | 0 | Hyper |
| NPR1 | cg14178748 | chr1 | 153679771 | Promoter, exon | -0.5832 | 0 | Hyper |
|  | cg26246928 | chr1 | 153679646 | Promoter, exon | -0.5160 | 0 | Hyper |
| PLIN1 | cg13632630 | chr15 | 89663098 | promoter | -0.2602 | 0.001343 | Hyper |
| SEMA3G | cg11137980 | chr3 | 52435210 | 5'UTR, exon | -0.6044 | 0 | Hyper |
| SORBS1 | cg02370232 | chr10 | 95415608 | intron | -0.6763 | 0 | Hyper |
|  | cg06282596 | chr10 | 95415722 | intron | -0.6528 | 0 | Hyper |
| FXYD1 | cg22783327 | chr19 | 35142354 | intron | 0.6777 | 0 | Hypo |
| MGLL | cg14476212 | chr3 | 127816348 | intron | 0.4838 | 5.33e-10 | Hypo |
| MYLK | cg09395562 | chr3 | 123782204 | intron | 0.5263 | 0 | Hypo |
|  | cg23726408 | chr3 | 123780033 | intron | 0.4852 | 4.62e-10 | Hypo |
| PLXNA4 | cg18301410 | chr7 | 132508133 | exon | 0.5448 | 0 | Hypo |
|  | cg02489094 | chr7 | 132487577 | intron | 0.5734 | 0 | Hypo |
| PDE2A | cg16640865 | chr11 | 72590514 | exon | 0.6494 | 0 | Hypo |
| SLIT3 | cg11855741 | chr5 | 169265185 | intron | 0.5467 | 0 | Hypo |
|  | cg12640653 | chr5 | 168756110 | intron | 0.4206 | 1.12e-07 | Hypo |
|  | cg16402822 | chr5 | 169100625 | intron | 0.5476 | 0 | Hypo |
|  | cg22631387 | chr5 | 169278984 | intron | 0.3472 | 1.52e-05 | Hypo |
