# Supplementary figures and images for: Discovered Key CpG Sites by Analyzing DNA Methylation and Gene Expression in Breast Cancer Samples
Source: Front Cell Dev Biol. 2022 Feb 1;10:815843. doi: 10.3389/fcell.2022.815843 (PMC8844453; doi:10.3389/fcell.2022.815843)

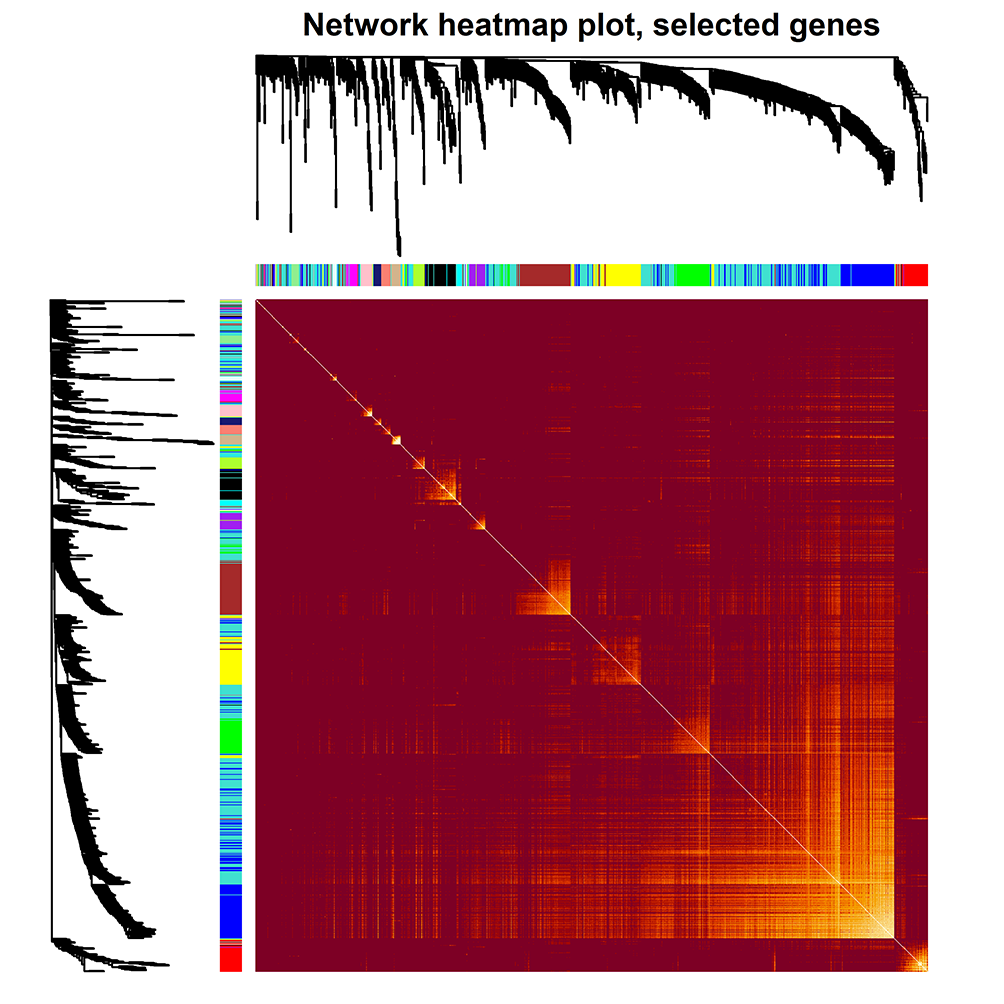

Supplement: Supplementary file 4 [file Image1.TIF]
